# Supplementary material for: Patterns of Intron Gain and Loss in Fungi
Source: PLoS Biol. 2004 Nov 30;2(12):e422. doi: 10.1371/journal.pbio.0020422 (PMC532390; doi:10.1371/journal.pbio.0020422)
Supplement: Table S1 — Also available at http://genes.mit.edu/NielsenEtAl/. (4.3 MB ZIP). [file pbio.0020422.st001.zip › NielsenEtAl/html/667.html]

AN5820.1.NCU08216.1.MG07384.1.FG06544.1


```
 CLUSTAL W (1.82) Multiple Sequence Alignments - Introns Inserted


Sequence 1: NCU08216.1	528 aa
Sequence 2: MG07384.1	533 aa
Sequence 3: FG06544.1	517 aa
Sequence 4: AN5820.1	535 aa
Alignment Length: 554 aa
Number Identitical Residues: 313 aa
Alignment Score (without introns) 13242


MG07384.1 	MAANTM--AARGAQSVTESATDLIGNTPLVRLNKIPQSLGIKAQVFGKLELMNTGGSVKD
NCU08216.1	MSGDTTNGAVERNPGLTMSATELIGNTPLVRLNRIPQSLGIECEVYGKVELFNAGGSVKD
FG06544.1 	MASQVP--HVLPRAATVHSATELIGNTPLVRLNKIPQSLGIECDVYVKPELFSAGGSVKD
AN5820.1  	MSSKTT---PTVPPVALDSITQHIGNTPLVRLNKLPQNLGINATVYAKLEYFNAGGSVKD
          	*:...             * *: **********::**.***:. *: * * :.:******

MG07384.1 	RIALRMIEEAEKEGRIKPGDTLIEPTSGNT2GIGLALVGAVKG~YKTIITLPEKMSAEKV
NCU08216.1	RIALRMIEEAEKEGRIKPGDTLIEPTSGNT2GIGLALVGAIKG~YKTIITLPEKMSAEKV
FG06544.1 	RIALRMIEEAEKSGRIKPGDTLIEPTSGNT2GIGLALVGAIKG~YKTIITLPEKMSAEKV
AN5820.1  	RIALRMIEEAERSGRIKPGDTLIEPTSGNT2GIGLALVAAVKG1YKTIITLPEKMSAEKV
          	***********:.***************** *******.*:** ****************

MG07384.1 	SVLKALGATIIRTPTQAAWDAPESHIGVAKRLLKEIPNSHILDQYSNPNNPLAHEHGTAE
NCU08216.1	SVLRALGATIIRTPTQAAWDSPESHIGVARRLLKEIPNSHILDQYTNPNNPLAHEFGTAE
FG06544.1 	SVLRALGATIIRTPTQAAWDAPESHIGVARRLEKELPNAHILDQYSNKDNPNAHEFGTAQ
AN5820.1  	SVLRALNATIIRTPNEAAYDSPESHIGVAKRLEKELPNAHILDQYGNENNPLAHELGTAQ
          	***:**.*******.:**:*:********:** **:**:****** * :** *** ***:

MG07384.1 	EIWAQTGGKITAVVAGAGTGGTISGLARGLRKHSKTVKVIAADPIGSILALPESLNDNGV
NCU08216.1	EIWKQTNGKVTAVVAGAGTGGTITGIARGLRKHNKDIKVIAADPVGSILAQPESLNDPNA
FG06544.1 	EIWEQTGGKITALVAGAGTGGTITGIARGLKKHNQDIKVIAADPHGSILALPELLNQEHI
AN5820.1  	EIWSQTKGQIKAIVAGAGTGGTITGLSRGLKKHNSNVQVIAADPQGSILALPAALNEEHA
          	*** ** *::.*:**********:*::***:**.. ::****** ***** *  **:   

MG07384.1 	NEPYKVEGIGYDFIPDVLDRELVDKWYKTEDRESFMYARRLIAEEGLLVGGSSGSAMAAM
NCU08216.1	TAPYKVEGIGYDFIPDVLDREFVDKWYKTEDRESFHLARRLIAEEGLLVGGSSGSAMAAM
FG06544.1 	NEGYKVEGIGYDFIPDVLDQHIVDKWYKTDDRESFHLARRLISEEGLLVGGSSGSAMAAM
AN5820.1  	NEPYKVEGIGYDFIPQVLDQHAVDKWYKTDDKESFQYARRLIAEEGLLVGGSSGSAIAAL
          	.  ************:***:. *******:*:***  *****:*************:**:

MG07384.1 	VRAVKDLNLGEDDVVVVVLPDSIRSYLS-~-KFADDDWLAANDLLLPTPDSSVNGDTLDS
NCU08216.1	IRAVKDYNLGKDDVVVVVLPDSIRSYLS-~-KFADDDWLAANGLLVTDDEIKTADEAKGT
FG06544.1 	LLAVKEYGFKKGDVVVVVLPDSIRSYLS-~-KFADDDWLAANNLLPVNGVESTVNGNGD-
AN5820.1  	VKAARDNMFKEGDVVVVILPDSIRSYLTK0IQFADDDWLAANDLLPSLPTEVILQSSNKQ
          	: *.::  : :.*****:*********:.  :**********.**               

MG07384.1 	PAVAEAQALHKSRARRASNPYGGATVRSLRLKPVTSVLADSKCSEAIETMRDKGFDQLPV
NCU08216.1	ATVKKQQETEQ---KKTDDGYAGATVRALRLKPVTSVLADSPCSEAIETMRDKGFDQLPV
FG06544.1 	--------------KKNSDPYEGATIAALRLKPVTSVGATSTCSEAIETMRDKGFDQLPV
AN5820.1  	T-------------QGQDDAFAGSKVSSLRLKPVTTVHSNVPCETAIEVMRDKGFDQLPV
          	.             :  .: : *:.: :*******:* :   *. ***.***********

MG07384.1 	VRDN--KLVGLVTLGNMLSYVSRGRVKTNSPVSDVMFDFGRLDEVITDPRDVEGAAKMST
NCU08216.1	LAPTGGKLVGLVTLGNILSFIQRGRATPATPVAKVMFDFSRLDEVVTDPR------KFDN
FG06544.1 	LSASGTKLVGLVTLGNLLSYISSGRATGQSSVSDVMFDFARLDEIVTDPR------EFAS
AN5820.1  	LAPSGRKLVGLVTLGNVLSRLTHNRATGRSPVADVMFDFRTISEVVTDPRDMG-LASLEP
          	:  .. **********:** :  .*..  :.*:.*****  :.*::****.    :.:  

MG07384.1 	AHG--------RKKRNFVEITMNTPLDVLSKFLEWNSAAIVTEKSGEV---SKPVAVVTK
NCU08216.1	NLK--------DKQRKFVDITMDTPLAALSKFLEWNSAAIVTEKTDNG---SKPVAVVTK
FG06544.1 	PINP-------GKRRQFVEITKDTPLTTLSKFFEWNSAAVVTEKSGDSKSLSKPIAVVTK
AN5820.1  	NQNGRDAPKPQTRSRKFVEITMDTSLSVLNRFFEWNSAAIVTERDEGG--TLRPVAVVTK
          	     .:.... : *:**:** :*.* .*.:*:******:***:        :*:*****

MG07384.1 	VDLLTWMMKELKA----
NCU08216.1	VDLLTYMLHQQSL----
FG06544.1 	VDLLTWMINKKL-----
AN5820.1  	VDLLTWMLHHKKNGASE
          	*****:*::.   .::.
```
